# Magnetoresponsive Functionalized Nanocomposite Aggregation Kinetics and Chain Formation at the Targeted Site during Magnetic Targeting

**DOI:** 10.3390/pharmaceutics14091923

**Published:** 2022-09-12

**Authors:** Sandor I. Bernad, Vlad Socoliuc, Daniela Susan-Resiga, Izabell Crăciunescu, Rodica Turcu, Etelka Tombácz, Ladislau Vékás, Maria C. Ioncica, Elena S. Bernad

**Affiliations:** 1 Romanian Academy-Timisoara Branch, Centre for Fundamental and Advanced Technical Research, Mihai Viteazul Str. 24, 300223 Timisoara, Romania; 2 Research Center for Engineering of Systems with Complex Fluids, Politehnica University Timisoara, Mihai Viteazul Str. 1, 300222 Timisoara, Romania; 3 Faculty of Physics, West University of Timisoara, Vasile Parvan Str. 1, 300222 Timisoara, Romania; 4 National Institute for Research and Development of Isotopic and Molecular Technologies (INCDTIM), Donat Str. 67-103, 400293 Cluj-Napoca, Romania; 5 Soós Ernő Water Technology Research and Development Center, University of Pannonia, Zrínyi M. Str. 18, 8800 Nagykanizsa, Hungary; 6 Department of Obstetrics and Gynecology, University of Medicine and Pharmacy “Victor Babes” Timisoara, P-ta Eftimie Murgu 2, 300041 Timisoara, Romania

**Keywords:** magnetoresponsive nanocomposite, functional coating, particle targeting, particle aggregation, stent targeting, nanomedicine

## Abstract

Drug therapy for vascular disease has been promoted to inhibit angiogenesis in atherosclerotic plaques and prevent restenosis following surgical intervention. This paper investigates the arterial depositions and distribution of PEG-functionalized magnetic nanocomposite clusters (PEG_MNCs) following local delivery in a stented artery model in a uniform magnetic field produced by a regionally positioned external permanent magnet; also, the PEG_MNCs aggregation or chain formation in and around the implanted stent. The central concept is to employ one external permanent magnet system, which produces enough magnetic field to magnetize and guide the magnetic nanoclusters in the stented artery region. At room temperature (25 °C), optical microscopy of the suspension model’s aggregation process was carried out in the external magnetic field. According to the optical microscopy pictures, the PEG_MNC particles form long linear aggregates due to dipolar magnetic interactions when there is an external magnetic field. During magnetic particle targeting, 20 mL of the model suspensions are injected (at a constant flow rate of 39.6 mL/min for the period of 30 s) by the syringe pump in the mean flow (flow velocity is Um = 0.25 m/s, corresponding to the Reynolds number of Re = 232) into the stented artery model. The PEG_MNC clusters are attracted by the magnetic forces (generated by the permanent external magnet) and captured around the stent struts and the bottom artery wall before and inside the implanted stent. The colloidal interaction among the MNC clusters was investigated by calculating the electrostatic repulsion, van der Waals and magnetic dipole-dipole energies. The current work offers essential details about PEG_MNCs aggregation and chain structure development in the presence of an external magnetic field and the process underlying this structure formation.

## 1. Introduction

Although PCI (percutaneous coronary intervention) is the most widely used treatment for those with atherosclerosis [1,2], it is invasive and has a high risk of restenosis—up to 16 percent for DES (drug-eluting stent) and 16–44 percent for BMS (bare metal stent) [2,3]. Furthermore, patients with advanced atherosclerosis commonly have complex plaques, which are more prone to rupture and result in coronary thrombosis, myocardial infarction, or other potentially deadly clinical events if not caught in time. It has been proven that nanoparticles are more beneficial for diagnosing atherosclerosis when targeted ligands are added [3,4].

Drug therapy for vascular disease has been promoted to inhibit angiogenesis in atherosclerotic plaques and prevent restenosis following surgical intervention [5,6,7,8]. However, when used systemically, anti-angiogenic or cytostatic medications frequently display unfavourable side effects. To enable the local application of the compounds, the chemicals were linked to balloons or stents [9,10] used in percutaneous transluminal angioplasty. This technique, in particular the use of drug-eluting stents laden with anti-proliferative agents, has resulted in restenosis rates below 10% [11,12].

To lower the risks of stent-related vascular injury or end-organ damage, our team has developed a technique for endothelial cell repair with direct targeting of the vascular wall under flow conditions.

Magnetic iron oxide nanoparticles and their nanocomposites, created with various functional coatings, are the most promising magnetic carriers for use in medicine [13,14,15,16,17], in particular in magnetically controlled tissue engineering [18] and cardiac regenerative medicine [19]. This is because essential requirements are met by them, including simple and non-toxic cellular uptake, superparamagnetic behaviour, the significant field-induced magnetic moments of multicore particles, good response to moderate magnetic fields, inherent ability to cross biological barriers, protection of the drug from rapid degradation in the biological environment, and a sizable surface area for conjugating targeting ligands. These features, together with characteristic sizes up to approximately 100 nm of the magnetic core of functionalized magnetic nanoparticle clusters dispersed in a biocompatible (usually aqueous) carrier, distinguish a category of ferrofluids as bio-ferrofluids [20,21]. These external stimuli nanosystems synthesized and experimented with in this paper were typical bio-ferrofluids and were designed to exploit the advantages offered by the high magnetic moment multicore particles (magnetic carriers) towards remotely controlled therapeutic performances in a stented artery.

Polyethylene glycols (PEGs) with the built-in EPR (enhanced permeability and retention) effect (increased permeability and retention) protected nanocomposite particle against unwanted protein corona formation, allowing them to enter cells and be employed in medicine. Site-specific drug delivery is the main challenge when delivering drug-carrying particles into the bloodstream [14,15].

Since it is stable, biocompatible, and hydrophilic, the polymer polyethylene glycol (PEG) has been the subject of extensive research for its potential in drug administration [22,23,24]. Furthermore, PEG reduces the immune system’s response to nanoparticles [25,26,27]. As a result, fewer nanoparticles are removed from the circulation, enhancing the concentration of nanoparticles at the target location, and lengthening the circulation duration [28,29,30].

Magnetic carriers can only partially reach their target areas before being recognized and expelled from the body by the mononuclear phagocytic system (MPS2) because of their propensity for aggregation and the presence of magnetic dipole-dipole interactions or van der Waals forces [31].

When an artery’s stents are targeted with magnetic carriers and then coated with an antirestenotic substance, in-stent restenosis is inhibited at drug dosages far lower than DESs. The following goals guided the present research:

1. To investigate the arterial depositions and distribution of PEG_MNCs following local delivery in a stented artery model in a uniform magnetic field produced by a regionally positioned permanent magnet.
2. To create a novel concept using permanent magnet systems to guide and target the functionalized nanocomposite around the stent to test the approach’s effectiveness for treating coronary heart disease (CHD).
3. PEG_MNCs aggregation or chain formation in and around the implanted stent (in the presence of the external magnetic field).

## 2. Materials and Methods

**Figure 1:**
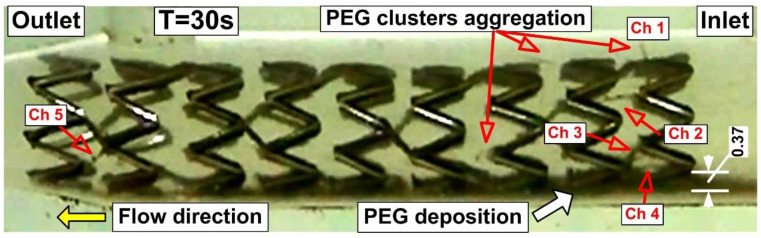
Flow-oriented large-scale magnetically induced aggregation of the PEG_MNC in the targeted region (red arrows) at the end of the injection period of 30 s. Magnetic cluster depositions correspond to the permanent magnet positions of 15 mm from the stent’s bottom wall. Ch 1 ÷ Ch 5–chain-like magnetic particles structure, generated in a different part of the stent geometry in the presence of the external magnetic field. Particle depositions on the bottom wall of the artery model in the stent inlet section (0.37 mm) at the end of the injection period of 30 s.

The competition between the magnetic force and the drag force exerted by the moving fluid, as well as the magnetic field gradient produced by the permanent external magnet and the ferromagnetic stent magnetic field is what causes the capture and deposition of the injected magnetic clusters in the flow stream (Figure 1).

**Figure 2:**
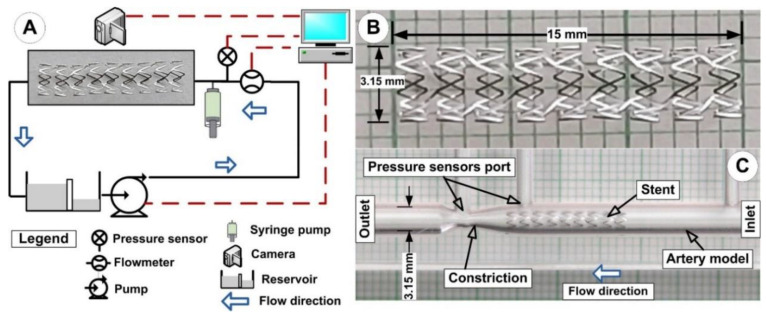
Implanted stent, particle targeting experimental setup. (**A**) The flowmeter, pressure sensors, suspension injection mechanism with a syringe pump, test section with magnetic stent model, reservoir, centrifugal pump, camera, and PC were all shown in the block diagram of the main recirculating flow loop. A suspension is injected using a syringe pump before the stent model enters part. (**B**) A general perspective of the stent geometry used and the distinctive dimension of the expanded stent. (**C**) Aerial view of the model artery showing the position of the stent.

**Figure 3:**
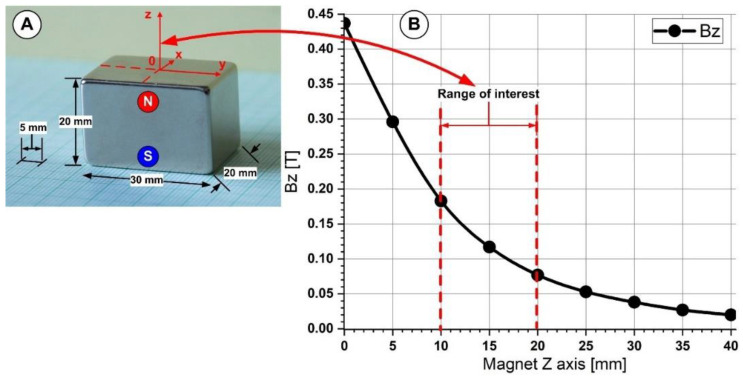
A magnetic field was generated by the NbFeB50 permanent magnet used in the experimental investigation. (**A**). The magnet’s dimension and axis association. A used permanent magnet has polarization along the *Z*-axis. (**B**) The figure depicts the magnetic field measured at various *Z*-axis positions with the F.W. Bell Gaussmeter, model 5080.

We calculated that the employed permanent magnet provided a consistent magnetic field of about 0.18 T and 0.08 T at the different locations from the magnet surface (10 mm and 20 mm), respectively, using magnetic field measurements (according to Figure 2 and Figure 3). Therefore, the magnetic force could be produced because the magnetic field stayed constant.

During magnetic particle targeting, 20 mL of the model suspensions are injected (at a constant flow rate of 39.6 mL/min for the period of 30 s) by the syringe pump in the mean flow (flow velocity is Um = 0.25 m/s, corresponding to the Reynolds number of Re = 232) into the stented artery model. The PEG_MNC clusters are attracted by the magnetic forces (generated by the permanent external magnet) and captured around the stent struts and the bottom artery wall before and inside the implanted stent (Figure 1). The evolution of the particle build-up is investigated using an image analysis technique.

All experimental measures are conducted in an air-conditioned chamber. The fluid is assumed not to be recirculated and to pass through the test section just once in the direction of flow when measurements are taken in an open circuit. This prevents the working fluid from heating up or changing its rheological properties throughout the measurements, with an injection time of 30 s. Furthermore, studies were conducted with a DC magnetic field. Direct current (DC) magnetic fields, in contrast to high-frequency AC (alternating current) magnetic fields, do not transfer thermal energy to magnetic nanoparticles or clusters injected in the test section.

### 2.1. Experimental Setup

The test portions are made of acrylic glass and are precisely shaped, with an interior diameter of 3.15 mm (Figure 2C). Our earlier publication [32] provided a thorough explanation of the stent targeting methodology, and our previous work [33] provided a complete description of the setup’s overall concept (Figure 2A). All flow tests were carried out in typical physiologic settings [34]. The main benefits of the acrylic glass model were its excellent transparency and ability to examine and evaluate the hemodynamic properties and the particle targeting process in the arterial stent segment.

The experimental setup demonstrated the removal of PEG_MNC from the flow flux and the deposition of the nanocomposite around the implanted stent’s geometry.

As previously mentioned in our articles [30] and articles [35,36], the implanted stent (Figure 2B) was made from magnetic 2205 duplex stainless steel (2205 SS).

It is significant to note that the Resolute Integrity Zotarolimus-Eluting Coronary Stent System (Minneapolis, MN, USA) and the stent used in the experimental setup have nearly identical geometrical characteristics (internal diameter of 3.15 mm, length of 15 mm, and strut thickness of 0.09 mm) (inner diameter of 3 mm, length of 15 mm, strut thickness of 0.09 mm).

### 2.2. Magnetic Field Generation

The central concept is to employ one external permanent magnet system, which produces enough magnetic field to magnetize and guide the MNP’s in the stented artery region, based on our prior findings [30,32,33].

We employed a Neodymium 50 type magnet (NdFeB50) with a maximum energy product (BxH) of 50 MGOe to create the magnetic field. According to our earlier research [30], the magnetic field produced by this neodymium magnet ranges from 0.44 T to 0.02 T or at a magnet distance of 0 mm (from the magnet surface) to 40 mm. We investigated the magnetic particle targeting in our studies for magnetic fields generated between 0.18 T and 0.08 T corresponding to the magnet position to the implanted stent between 10 mm and 20 mm (along with the magnet z axis—Figure 3).

### 2.3. Blood Analogue Fluid, Preparation, and Rheological Properties

In our studies, the carrier fluid (CF) was glycerol-water solutions, which were made by combining estimated weights of glycerol and distilled water and having a density (1060 kg/m^3^) that is identical to that of blood [30]. This CF made it easier for the experimental investigations to accurately reproduce the rheological behaviour of the fluid flow at the spot of the implanted stent. The PEG_MNC dispersed in distilled water, with a 0.1 percent mass concentration, was combined with a carrier fluid (CF) to create the model suspension of magnetic carriers utilized in tests.

**Figure 4:**
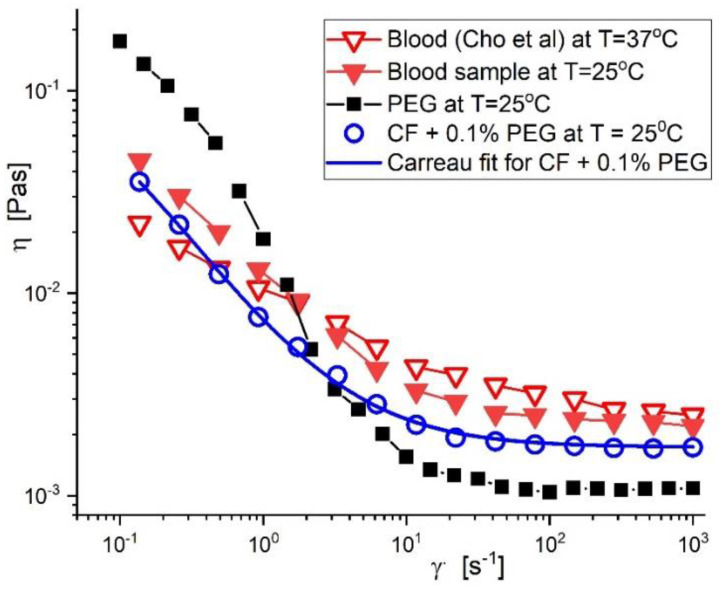
Viscosity curves for blood (values from literature and healthy volunteer), PEG −coated functionalized nanocomposite (PEG_MNC), and model suspension fluid (carrier fluid + 0.1% PEG_MNC).

The agreement between the model suspension’s viscosity curve at T = 25 °C, the blood sample (obtained from a 38-year-old female volunteer in good health), and the results presented in the literature [37] is shown in Figure 4. During these investigations, we did not use any blood samples. Instead, we used one example from the existing databases in our institution. The data from the databases were used in a comparative sense with data from the literature and prepared blood mimicking fluid.

The Carreau model with four pertinent parameters can be used to define the rheological values for the model suspensions, as shown in Table 1. (Equation (1)).

(1) $$\eta \left(\dot{\gamma }\right)=\eta_{\infty }+\left(\eta_{o}-\eta_{\infty }\right){ [1+{\left(C\dot{\gamma }\right)}^{2}]}^{-p}$$

in which *C* [s] represents the Carreau constant (the value of the slope of the viscosity curve in the log-log scale at high values of the shear rate $\dot{\gamma }$, *p* [-] is the Carreau exponent, $\eta_{o}$ is the viscosity at infinitely low shear rates, and $\eta_{\infty }$ [Pas]- viscosity at infinitely high shear rates.

## 3. Results

### 3.1. Synthesis and Characterization of the PEG-Functionalized Magnetic Nanocomposite Clusters (PEG_MNC)

Our earlier work [22,23,30] goes to great length about the synthesis of PEG-coated magnetite nanoparticles, the magnetic and colloidal characteristics of the core-shell, their biocompatibility, and their potential for use in biomedicine. Briefly, we prepared the PEG-coated MNCs by the oil-in-water miniemulsion. As presented in our previous work [30], PEG (1.795 g), which functions as a surfactant, was added to an aqueous solution with a toluene-based ferrofluid (0.5 wt% Fe3O4). PEG molecules arranged themselves with the polar end in the water phase and the nonpolar end in the oil phase, forming micelles when they were present. The newly formed droplets included toluene-dispersed magnetic nanoparticles. First, the two-phase mixture was homogenized using an ultrasonic finger U.P. 400S for 2 min to produce a stable miniemulsion. The second step involved evaporating the toluene organic phase in an oil bath at 100 °C while being magnetically stirred at 500 rpm. After being cleaned of any excess reactants with the methanol-water solution (50 mL), the magnetic clusters were redispersed in distilled water. The PEG-coated MNCs were dispersed in an aqueous carrier (blood analogue fluid). Analog was used as a carrier fluid (CF) for blood and was made from glycerol-water solutions by combining distilled water and glycerol at predetermined weights.

**Figure 5:**
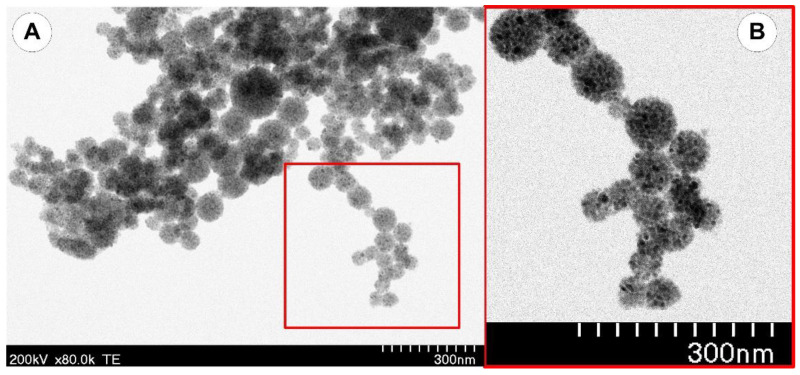
TEM image of the magnetic nanoclusters coated with PEG (**A**). Detail of the spherical clusters (**B**).

**Figure 6:**
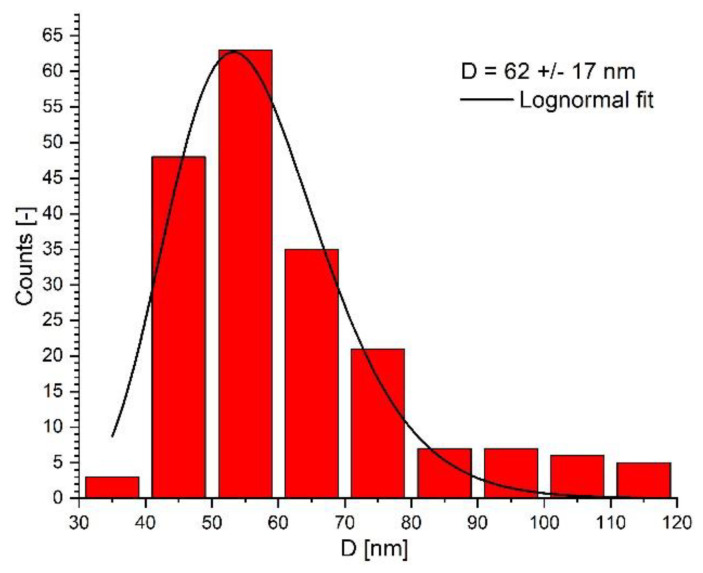
TEM size distribution and lognormal fit of the PEG−coated. The TEM size distribution was obtained by analysis of the TEM micrograph shown in Figure 1.

A Hitachi HD2700 microscope was used for the electron microscopy (TEM) investigation into the morphology of the magnetic clusters. The magnetic nanoparticles highly packed into spherical clusters (Figure 5) with diameters of 40 to 120 nm are depicted in the representative TEM images of the PEG_MNC in Figure 1. The average diameter of the PEG-coated clusters (size range 40–120 nm), according to TEM image analysis of micrographs is 62 ± 17 nm (Figure 6).

The PEG_MNC diameter distribution was obtained using ImageJ [https://imagej.nih.gov/ij/ (accessed on 3 August 2022)]. A number of 195 PEG_MNCs from three TEM images were measured. Figure 6 is presented the magnetic nanoparticles’ diameter distribution. The diameter distribution shows positive skewness, with a 62 nm mean diameter. Due to the clustering during the TEM sample preparation evaporation process, the question remains whether the MNCs are soldered within the clusters or not.

**Figure 7:**
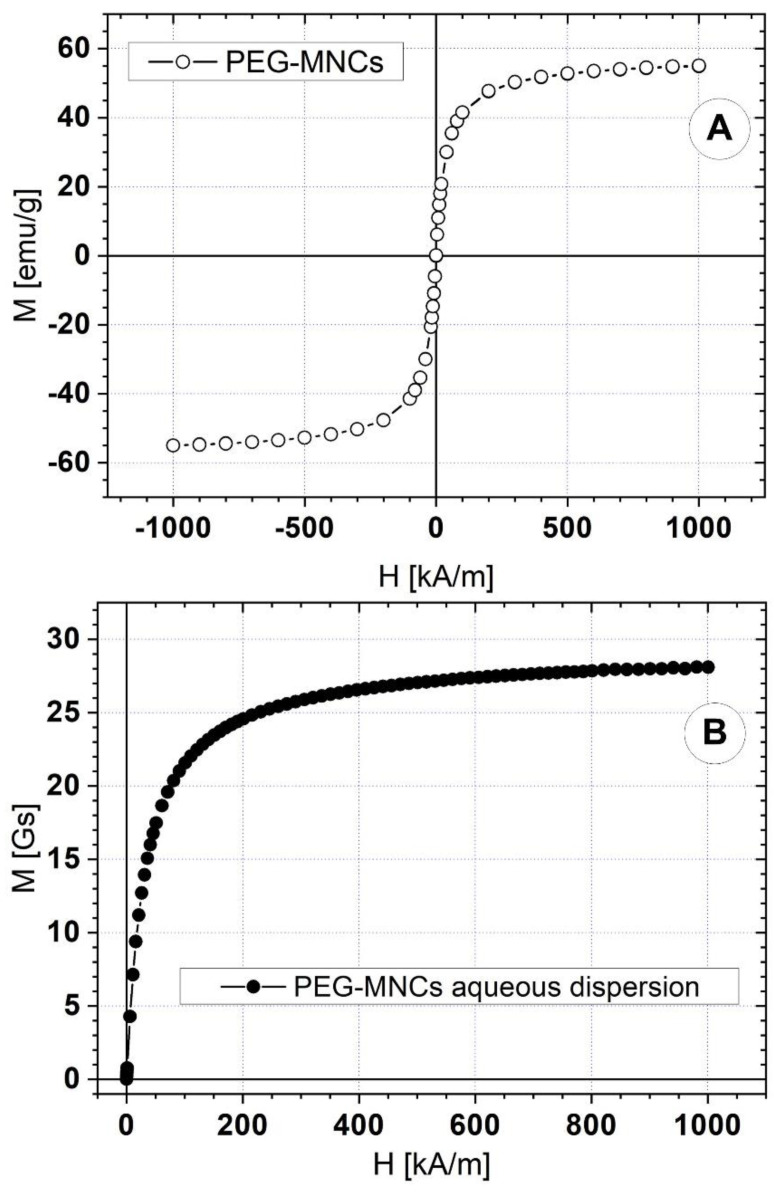
Magnetization curves for (**A**) a sample of dry PEG-MNCs, and (**B**) PEG_MNCs aqueous dispersion at room temperature (25 °C).

A vibrating sample magnetometer (VSM 880-ADE Technologies, Massachusetts, MA, USA) with a field range of 0 kA/m to 1000 kA/m was used to measure the magnetic characteristics of the PEG-coated clusters. Figure 7 shows the PEG_MNCs’ (A) and its aqueous dispersion (B) magnetization curves at room temperature.

Both samples show superparamagnetic behaviour. The PEG − MNCs have zero remanent magnetization and 55 emu/g saturation magnetization. The PEG − MNC aqueous dispersion has 28 Gs saturation magnetization.

### 3.2. Rheological Properties of the PEG_MNC Aqueous Dispersion

An *Anton Paar* Physica MCR 300, Graz, Austria rheometer was used. The magnetorheological cell (plate-plate geometry PP20/MRD/TI-SN18581), has a diameter of 2R = 20 mm, and a gap fixed at h = 0.2 mm, was used for the rheological measurements in this article. The sample layer between the plates is subjected to a perpendicular magnetic field in this cell. The magnetic flux density of the applied magnetic field is determined using a Hall probe inserted under the bottom plate of the MR cell.

Numerous cardiovascular illnesses have mechanical blood artery wall behaviour and blood flow characteristics contributing to their onset and progression [31]. Blood exhibits non-Newtonian fluid behaviour in the small/capillary arteries while acting similarly to a Newtonian fluid in the major arteries [38]. Massive viscosity fluctuations in these blood arteries indicate the pseudoplastic nature of flow at low shear rates ($\dot{\gamma }<100s^{-1}$) [39].

#### Viscosity Curves of PEG_MNC Aqueous Dispersions

**Figure 8:**
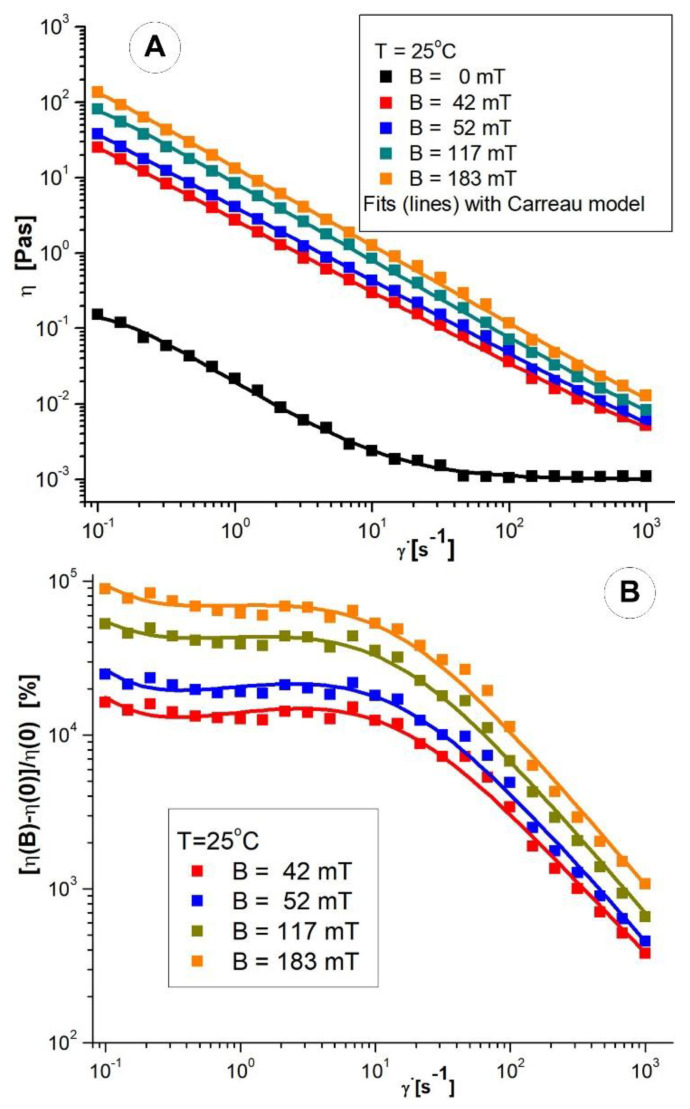
(**A**) The model suspension viscosity curves at T = 25 °C in the presence and absence of the magnetic field are shown; (**B**) MV effect as a function of shear rate at different values of the magnetic flux density, at T =25°C.

Viscosity curves at different values (*B* = 0, 42, 52, 117, 183 mT) of applied magnetic induction were measured in the range 0.1–1000s^−1^ of shear rates, at temperature value, *T* = 25 °C (Figure 8).

A shear-thinning behaviour of the PEG_MNC suspension is observed, both in the absence and in the presence of the magnetic field. The shear −thinning behaviour character is accentuated in the magnetic field’s presence when the magnetic interactions’ intensification induces agglomerations of magnetite clusters. As the shear rate increases, these cluster agglomerations are progressively destroyed, and as a result, the suspension’s viscosity decreases.

The relative increase in viscosity induced by the magnetic field (magneto viscous effect-MV) is already significant when applying weak fields, and this phenomenon tends to saturate as the magnetic field intensifies [40,41].

The *η* = *f*($\dot{\gamma }$) data were correlated with the Carreau model [42], Equation (1).

The values obtained for the fit parameters are listed in Table 2. As expected, in the presence of the magnetic field, the values of the fit parameters *η*_0_, *C*, and *p* increase with the intensification of the field.

The influence of shear rate on the MV effect at different magnetic flux density values at T = 25 °C is represented in Figure 8B. It is observed that the effect of MV on the range of low shear rates ($\dot{\gamma }=\left(0.1-10\right){\;s}^{-1}$)) is almost independent of the shear rate but significantly decreases at high speeds as cluster agglomerations are destroyed. The MNC agglomerates are destroyed for higher shear rates, and the MV effect decreases. The observed magnetoviscous behaviour is a consequence of the multicore nature of the magnetic component resulting in a high induced magnetic moment of particles, favouring their structuring in a magnetic field. The PEG-coated magnetic nanoclusters of 62 nm mean size suspended in a glycerol-water carrier (mass concentration 50 mg/mL) show a very high MV effect of about 10^4^%. Commercial bio-ferrofluids with multi-core magnetic particles of hydrodynamic diameters of 50, 100 and 200 nm were investigated in [40]. They manifested a significant, up to 1500x increase, in the effective viscosity in the magnetic field already at a significantly lower mass concentration of multicore magnetic particles of about 25 mg/mL. At the same time, in the case of highly concentrated aqueous ferrofluids with predominantly single core (less than 10 nm) magnetite nanoparticles, the magnetoviscous effect is very small and manifests only up to a 50% increase in viscosity [41]. The careful design of the size and, implicitly, of the magnetic moment of nanoclusters is essential to meet the optimal biomedical size window (50–150 nm) requirements and the need for moderate magnetic fields to control the movement of particles.

Magnetorheology evidences the possibility of remote control by already a moderate magnetic field of the motion, interaction and collection of PEG_MNC particles in the required regions of the stent, a condition of efficient magnetic targeting.

### 3.3. PEG_MNC Sedimentation

**Figure 9:**
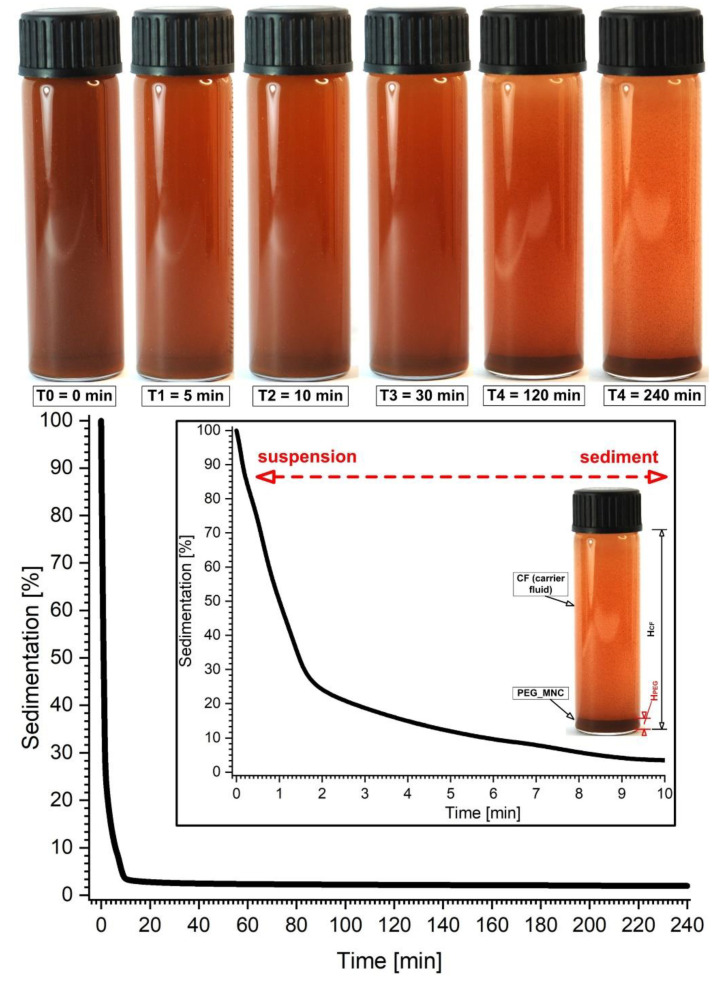
Sedimentation profile as a function of time for PEG_MNC suspension. Detail regarding sedimentation profile in the first 10 min. CF–carrier fluid; PEG_MNC-PEG-coated magnetic nanoclusters.

The sedimentation profile of the PEG_MNC nanoclusters was also investigated during this experiment. For 240 min, the sedimentation profile was captured as a function of time. During the first 10 min, the PEG-coated nanocluster’s sedimentation was visible, and after that, a steady state was attained (Figure 9). In other words, the PEG_MNC suspension’s dispersion stability was sufficient for our experiment (suspension injection time in the targeted region range between 20 to 120 s).

### 3.4. PEG_MNC Delivery at the Targeted Site

Flow-mediated particle transport achieves the PEG_MNC delivery at the targeted site in the present experiment.

**Figure 10:**
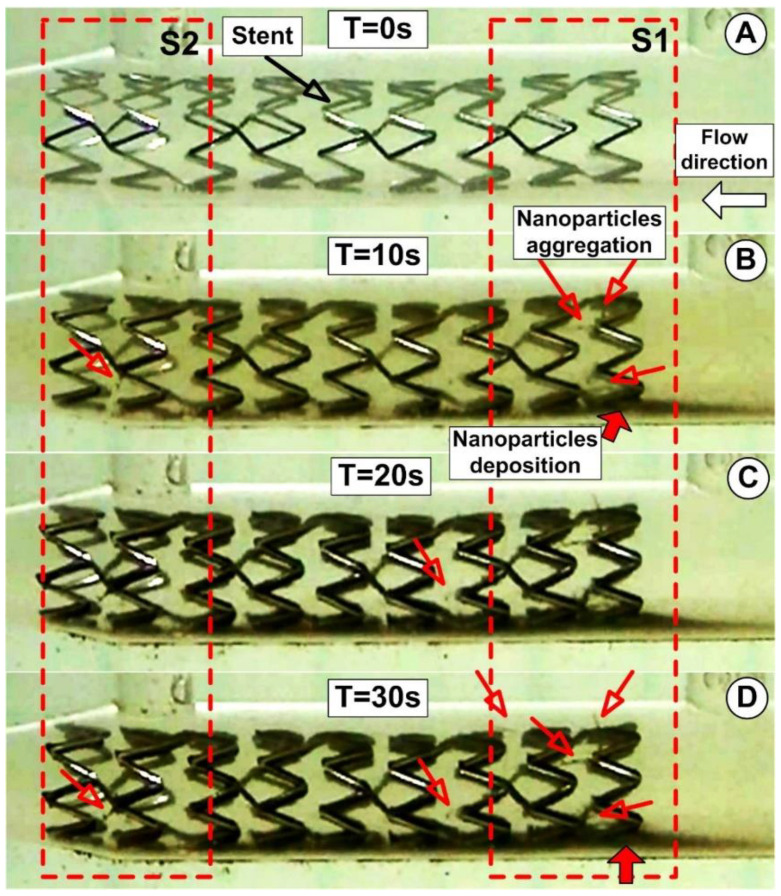
Magnetically induced large scales aggregation of the PEG_MNC around the targeted magnetic stent. Aggregation size evolution during different time steps of the injection period of 30 s. S1 and S2 regions where chain-like magnetic particles structure are generated (red arrows).

The evolution of the functionalized magnetoresponsive clusters build-up is acquired at a frame rate of 30 frames/s. The MP’s deposition and the magnetically induced chain length are investigated at the end of the injections period (after 30 s) (Figure 10D). These parameters are estimated as a function of the initial PEG_MNC quantity dispersed in the model suspensions and the injections period.

The PEG_MNC accumulation for the various injection time steps near the stent struts for the fixed magnet position is shown in Figure 10 (magnet position at 15 mm from the stent lower part and 18.15 mm from the upper part). As shown in Figure 10, the circulating magnetic clusters eventually completely encircle the stent struts. It is crucial to note that the magnetic cluster depositions are uneven, more robust at the last strut ring in the stent exit section, and more uniform on the first strut ring (in the stent inlet region). This occurs because the geometry of the stent and the strength of the applied external magnetic field affect local magnetic cluster deposition. Figure 9 illustrates this situation clearly, showing how the exposed portion of the struts from the distal end of the stent was typically not well covered by magnetic clusters. The cluster deposition around the stent struts is significantly influenced by local hemodynamics. The near-wall residence duration was prolonged, and the magnetic particle deposition was improved by the presence of the recirculation flow around the struts. The balance between the hydrodynamic force produced by the angular velocity of the recirculation region, the intensity of the magnetic field produced by the ferromagnetic stent, and the superimposed external magnetic field also contributes to the coverage of the struts with magnetic clusters. In particle deposition, the magnet position is crucial. The collection of particles in the bottom part is more noticeable than the accumulation of particles in the top part of the stent because the magnet is closer to the stent’s lower part. The syringe’s exit part moved at the same speed as the working fluid through the artery model during the model’s suspension injection, which was carried out at a constant flow rate of 1.5 mL/s. Given the proximity of the PEG suspension injection point to the stent (19 × stent diameter ≅ 60 mm), the PEG_MNC begins to deposit around the proximal stent struts and at the stent inlet section nearly within one second (Figure 10B).

As observed in Figure 10D, the accumulation of particles in this location is more prominent than the accumulation of particles in the top part of the stent because the magnet is closer to the stent’s bottom part (around the stent elements). Additionally, using a single magnet causes increased particle collection on the bottom wall of the artery due to the magnet’s position on the stent.

Using an image-processing application (ImageJ, https://imagej.nih.gov/ij/ (accessed on 3 August 2022)), the thickness of the particles on the artery’s lower wall was measured to quantify the PEG_MNC deposition at the end of the injection period of 30 s. (Figure 10D).

### 3.5. PEG_MNC Aggregation and Chain Formation Inside the Expanded Stent

The near-wall residence duration was prolonged, and the magnetic particle deposition was improved by the presence of the recirculation flow around the struts. However, it is crucial to note that the magnetic cluster depositions are uneven, more robust at the last strut ring in the stent exit section, and more uniform on the first strut ring (in the stent inlet region).

The acceleration of the particles in the direction of the magnetic field source is caused by the magnetic force, which is produced by an external magnetic field. Therefore, the magnetic flux concentrations close to the target location are more significant than 110 mT (120 mT at the distance of 15 mm) (Figure 3), sufficient to cause particle saturation.

**Figure 11:**
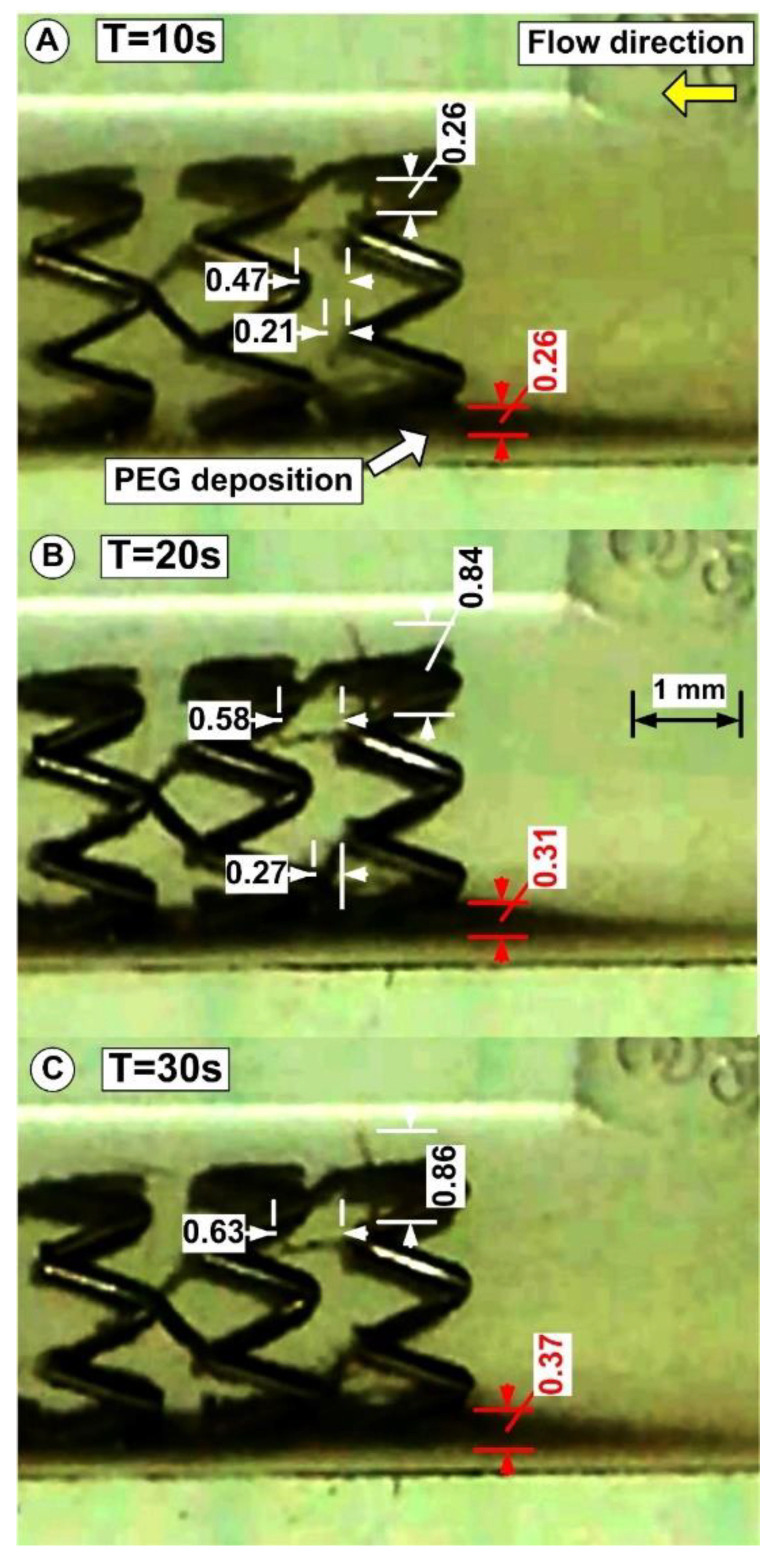
Detail regarding the chain-like particle structure evolution during injection time of 30 s. Data shows chain structure development and growth for chains Ch 1, Ch 2, and Ch 3 (according to Figure 9). Additionally, the figure presents the PEG_MNC deposition evolution during the injection period of 30 s (red arrows). All presented data are in mm.

In our experiment, magnetic particles create chain-like structures in a stented arterial model (Figure 11). The chains are usually oriented according to the applied external magnetic field perpendicular to the flow direction. In our case, the exception is chained Ch 2 and 4, where the geometry of the stent affects how the chains develop. Due to the strong magnetic field, the parabolic flow profile does not bend the shape of the chains.

The chain length variations corresponding to different injection times are presented in Table 3.

### 3.6. Suspension Model’s Aggregation Process in the Magnetic Field

At room temperature (25 °C), optical microscopy of the suspension model’s aggregation process was carried out in the external magnetic field. The microscope bench was modified to accommodate a field generator based on permanent magnets that produced a 100 mT magnetic field and had an optical cell with a 0.1 mm thickness.

Randomly dispersed particles become magnetized by a perpendicular applied field due to field-induced chaining.

**Figure 12:**
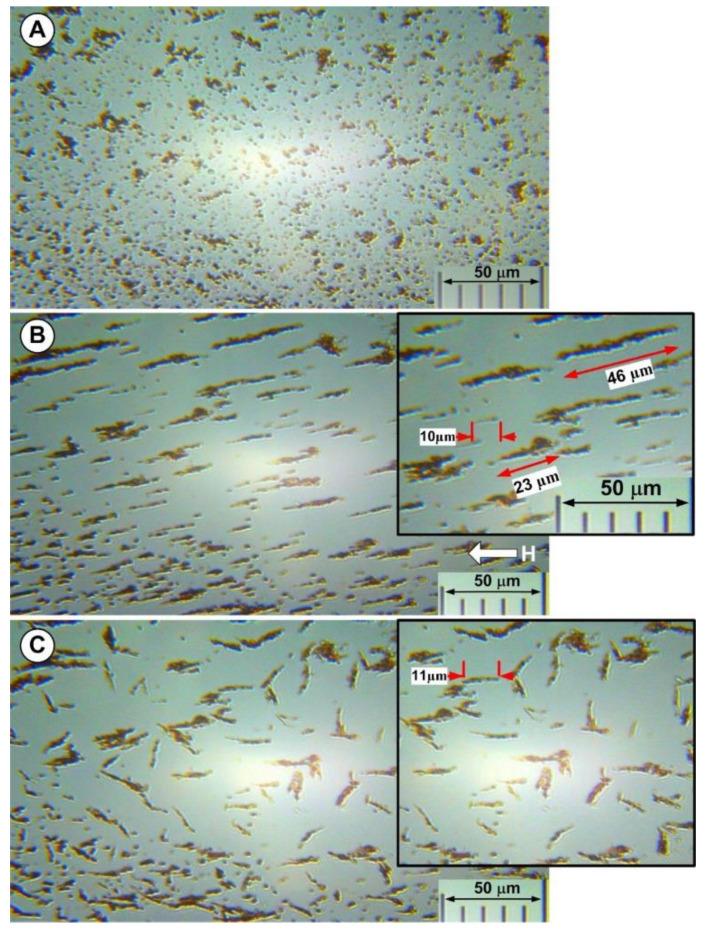
(**A**) PEG_MNC nanocomposite in carrier fluid without the magnetic field (optical microscopy). The figure shows the initial state with a homogeneous nanoparticle suspension when the magnetic field is switched on (t = 0); (**B**) aggregation of the PEG_MNC nanocomposite in carrier fluid under the action of the externally applied magnetic field of intensity H = 110 mT. Detail regarding the length and thickness of different chains generated. (**C**) Evolution of the PEG_MNC aggregate after turning off the magnetic field. Detail shows that the size of nanoparticle aggregates decreases after the magnetic field is turned off.

The microscope pictures of the PEG_MNC suspension before magnetic field application (Figure 12A) and the following 5 s of the magnetic field application are shown in Figure 12B. According to the optical microscopy pictures (Figure 12B), these particles form long linear aggregates due to dipolar magnetic interactions when there is an external magnetic field.

Considering that the average sizes of the multicore particles (as determined by DLS) are 102 ± 28 nm (size range: 70 ÷ 150 nm), these significant structures generated in the range of several micrometres (formed under magnetic fields) assume that the behaviours found might lead to the characteristics of the MVE discussed in the previous chapter.

To understand how the strength and length of the external magnetic field affect the size of the chain-like structures, it is crucial to study how the formations arise.

The spindles in the final stages of the process have an average thickness of ≅ 2 µm and a length of tens of microns (Figure 12B).

### 3.7. Colloidal Stability of the PEG_MNCs

The colloidal stability of the PEG_MNCs was investigated. For this purpose, an MNC aqueous dispersion (MNC-AD) was prepared using one stage of distilled water. Then, the MNC-AD was vigorously stirred using ultrasonication in several successive steps. After each ultrasonication step (~1000 J/step in 10 mL MNC-AD), most MNCs sediment within about one hour, leading to a diluted turbid supernatant.

The hydrodynamic diameter and zeta potential were measured with a Malvern Zetasizer Nano instrument. Dynamic light scattering provides three crucial details about the final PEGylated NP [43,44]: NP size, zeta potential (details on the NPs’ colloidal stability and surface coating), and size distribution. The Malvern Zeta Sizer Nano Series (operated at a scattering angle of 173°) was used for the DLS measurements (1 mL of particle suspensions were deposited in a 10 × 10 mm quartz cuvette). A He-Ne laser was also included in the setup. These clusters’ average sizes, as determined by DLS, are 102 ± 28 nm (size range: 70–150 nm).

**Figure 13:**
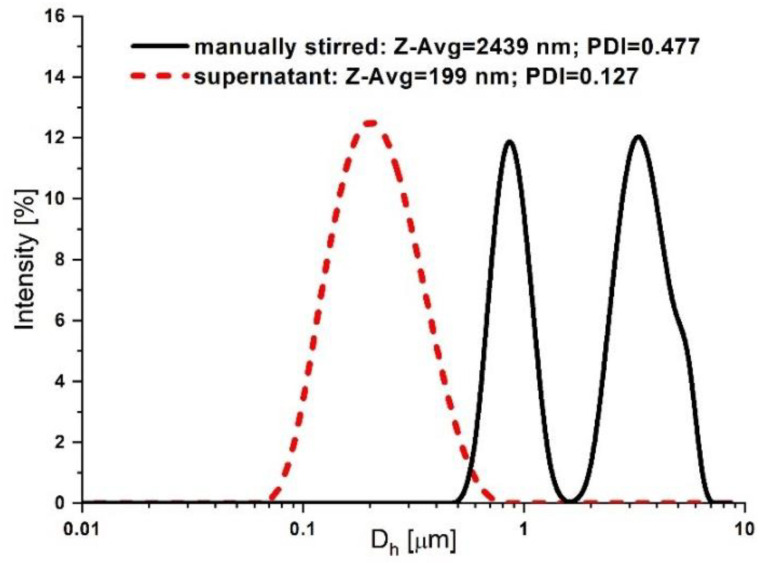
DLS size distribution by the intensity of the manually stirred PEG_MNC sample and the supernatant.

The DLS size distribution by the intensity of the MNC-AD did not change significantly after six successive ultrasonication steps and was the same as after vigorous manual stirring. Figure 13 presents the size distribution by the intensity of the manually stirred sample and the supernatant. The Z-average hydrodynamic diameter in the manually agitated sample was 2439 nm (PDI = 0.477) with an apparent two-mode distribution (~0.9 μm and ~3 μm). Therefore, most MNCs are soldered in large permanent clusters due to bridge interactions between polymer shells (Figure 12B). Even in the supernatant, the measured Z-average hydrodynamic diameter was 199 nm (PDI = 0.172) which, compared to the TEM 62 nm diameter, indicates a small degree of permanent clustering of the MNCs.

The ζ-potential of the MNCs measured in the supernatant was −25 mV.

The MNC-AD was investigated by optical microscopy. Vigorous manual stirring of the sample was conducted before visual cell sample preparation. Figure 12 presents sample images before, during, and after a 110 mT external magnetic field application. Before the magnetic field application, the DLS data shows MNC clusters with sizes of 1 ÷ 10 μm (Figure 12A). Under the action of the external magnetic field, filed-oriented long (~32 μm) MNC cluster filaments are formed (Figure 12B). After removing the magnetic field, the filaments become randomly oriented (Figure 12C) but do not spontaneously dissolve. The filaments are dissolved, however, after mild ultrasonication or manual stirring, thus indicating that the magnetic dipole-dipole attraction potentiated soft attraction between the MNC clusters during the external field.

### 3.8. Colloidal Interactions among the PEG_MNCs

The colloidal interaction among the MNC clusters was investigated. The electrostatic repulsion and van der Waals and magnetic dipole-dipole attraction were calculated.

The surface separation dependence (*x*) of the electrostatic repulsion energy is [45,46]:

(2) $$U_{el}\left(x\right)=64\cdot \pi \cdot \varepsilon_{r}\cdot \varepsilon_{0}\cdot \gamma^{2}\cdot {\left(\frac{k_{B}T}{\nu \cdot e}\right)}^{2}\cdot \frac{R^{2}}{2\cdot R+x}\cdot e^{\left(-\frac{x}{l_{D}}\right)},$$

where $\gamma =Tanh\left(\frac{\nu \cdot e\cdot U_{Z}}{4\cdot k_{B}T}\right)$ and $l_{D}=\sqrt{\frac{\varepsilon_{r}\cdot \varepsilon_{0}\cdot k_{B}T}{2\cdot N_{A}\cdot e^{2}\cdot I_{S}}}\;$ is the Debye length. *R* is the MNC radius; *U_z_* is the ζ-potential (measured −25 mV), *ν* is the electrolyte charge number (assumed to be ν = 1), *ε*_0_ is the vacuum permittivity, *ε_r_* = 78.4 is the relative permittivity of water, e is the electron charge, *k_B_* is Boltzmann’s constant, *T* is the temperature (T = 300 K), *N_A_* is Avogadro’s number (N_A_ = 6 × 10^23^ 1/mol). *I_s_* the water ionic strength (expressed in mol/mc).

To evaluate Equation (2), the ionic strength of the water needs to be determined. In principle, the water ionic strength can be calculated if complete knowledge of the ion content can be obtained. Unfortunately, we could only determine some of the water’s ionic content using atomic mass spectrometry at AQUATIM S.A., Timisoara, Romania.

Moreover, due to storage conditions, our distilled water was kept in contact with the atmosphere; therefore, dissolved carbon dioxide is very plausible, but we could not measure it. Therefore, to compensate this missing information, we used the AQION 7.3.3 application [www.aqion.de (accessed on 27 July 2022)] to correct the ionic content of the water to obtain the measured water electrical conductivity (*E_c_* = 3 μS/cm) and pH (pH = 7.4) and use the resulting ionic strength: *I_s_* = 0.0257 mol/m^3^.

The Van der Waals interaction energy among identical MNCs is [47]:

(3) $$u_{vdW}\left(z\right)=-\frac{A}{12}\cdot \left(\frac{2R^{2}}{{\left(2R+z\right)}^{2}}\right.+\frac{2R^{2}}{{\left(2R+z\right)}^{2}-4R^{2}}+\left.\ln\left(\frac{{\left(2R+z\right)}^{2}-4R^{2}}{{\left(2R+z\right)}^{2}}\right)\right),$$

The Hamaker constant *A* for magnetite in water is 20–40 zJ [47]. For estimation purposes, we shall use the lower value *A* = 20 zJ to account for the surfactant’s presence in the MNCs’ structure.

The calculation of the magnetic dipole−dipole attraction energy among MNCs was made using the model developed by Socoliuc and Turcu [48]. In brief, the model describes the magnetic dipole-dipole interaction energy among MNCs, assumed as superparamagnetic clusters with zero remanent magnetization.

**Figure 14:**
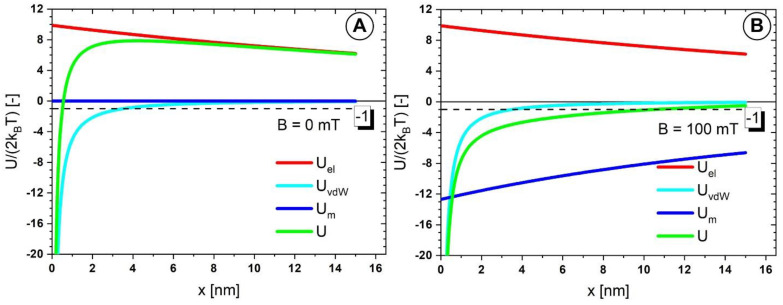
Electrostatic repulsion energy (red line), Van der Waals energy (blue), magnetic dipole-dipole energy (cyan), and resultant energy (green line) for (**A**) B = 0 mT, and (**B**) B = 100 mT.

Figure 14 is presented the surface−to−surface (*x*) dependence of the interaction energy (in k_B_T units) among two 62 nm diameter MNCs. The red line is the electrostatic repulsion energy (Equation (2)), the blue line is the van der Waals energy (Equation (3)), and the cyan is the magnetic dipole-dipole attraction [47]. The green line is the resultant energy U. If U/(2k_B_T) is below −1, the MNC aggregates are thermodynamically stable.

At B = 0 mT, the electrostatic repulsion dominates the interaction. No spontaneous clustering should occur provided that the shell thickness is at least 0.5 nm. For x > 0.5 nm the resultant energy exceeds the stability threshold of −1 (Figure 14A).

At B = 100 mT, the magnetic dipole-dipole interaction is the attracting driving force. Once the MNCs close below the 10 nm range (much larger than the PEG polymer shell thickness) stable clusters form. If the shell is soft, the magnetic attraction presses the MNCs and thus, after the field removal, the Van der Waals attraction dominates the interaction, similarly to a “click” connection.

This explains, on the one hand, the remanence of the MNC cluster needles after the field switch-off (Figure 12C) and, on the other hand, the ease with which these needles are dissolved after mild manual stirring.

The electrostatic repulsion for MNCs clusters is strong enough to allow rapid dissolution after ultrasonication. The fact that this was not possible is proof for the assertion that the MNCs are soldered in the clusters, most likely due to bridge interactions among PEG shells or collective engulfment in PEG of multiple MNCs.

## 4. Discussion

### 4.1. Clinical Perspective

The current study shows the viability of a novel site-specific drug delivery approach based on the uniform-field-induced magnetization effect and its application for drug-loaded magnetic particles targeting stented arteries. PEG_MNCs, which were employed in these proof-of-concept tests, were created with the following set of qualities that make a practical and secure magnetic drug carrier; due to the numerous small-sized iron oxide nanocrystals incorporated into each particle, the multicore particles exhibit the following properties: (1) magnetic solid responsiveness in the absence of magnetic memory; (2) suitability for intravascular delivery and targeted drug delivery; (3) functionalization with polymer, polyethylene glycol (PEG), to enhance biocompatibility with cells, enable long-circulating properties for systemic delivery, and reduce particle accumulation in the body.

The current findings also offer several significant insights into the mechanisms driving the homogeneous magnetic fields of magnetic particles targeting magnetic stents. The present work also offers essential details about PEG_MNC aggregation and chain structure development in an external magnetic field and the process underlying this structure formation. Predicting the therapeutic success of the employed treatment requires extensive knowledge of the drug delivery process.

The medication targeting approach using functionalized magnetic nanoparticles is particularly appealing for clinical applications since it could avoid restenosis and other problems following invasive operations, such as plaque removal or stent placement.

### 4.2. Limitations

The precise and repeatable targeting of magnetic nanostructures by an external magnetic field and the improved production of magnetoresponsive nanocomposite particle to enhance nanoparticle properties such as their shape, surface charge, size, ligands, and magnetic performance remain problems in drug targeting applications.

Since the magnetic field’s force must outweigh the hydrodynamic (drag) force and the shear effects produced by the running blood cells, magnetic capture of nanoparticles at flow conditions typical of the medium and large vessels may be more challenging to accomplish. The magnetic field gradients and the flow dynamics consequently govern the behaviour of magnetic particles in circulation and the effectiveness of their aggregation in addition to the nanoparticle features [49].

Our finding provide proof of particle depositions supporting the steady nature of blood flow. Additionally, the blood flow’s increased velocity causes the hydrodynamic drag force to increase, which causes variances in particle depositions. Therefore, the significance of including more accurate arterial blood flows (pulsatile flow field) in the experimental research of magnetic drug targeting is mandatory.

## 5. Conclusions

Other effects on magnetic nanoparticle use in biomedicine result from the creation of linear aggregates. Aggregates will drastically alter the system’s magnetophoretic mobility as they form. When paired with the acicular form and size of the aggregates, larger particles’ generally greater magnetophoretic mobility may drastically change the behaviour of the particles for applications in magnetically targeted drug delivery [50].

The main reason NPs aggregate is because the attraction of the particles to one another is stronger than the attraction of the solvent [51,52].

The Derjaguin-Landau-Verwey-Overbeek (DLVO) theory [53] states that NPs with high surface energy have a propensity to assemble. The electrostatic repulsive potential and the Van der Waals attraction potential are related to the interaction potential for spherical NPs [54]. PEG lessens Van der Waals attraction and lowers the surface energy of NPs [55,56].

By widening the steric gap between them and boosting hydrophilicity by ether repeats creating hydrogen bonds with solvent, PEG reduces the attraction between NPs. In addition, the particle size can be changed using PEGylation, which has additional advantages.

Long-ranged magnetic dipolar interactions that arise in the presence of a field in this situation would predominate over colloidal ones (Van der Waals, steric, and electrostatic forces).

The electrostatic repulsion energy, Van der Waals, and magnetic dipole-dipole attraction were calculated in the absence and presence of the external magnetic field (magnetic field intensity of B = 100 mT) to examine the colloidal interaction between the PEG_MNC clusters.

This research investigated the magnetic separation and field-induced aggregation of multicore IONPs with an average metal oxide multicore size of 62 ± 17 nm, coated by a PEG monolayer.

Aggregation might be seen as a field-induced phenomenon as a result. The magnetic nanoparticle chains exhibit very elongated structures that substantially outweigh the individual nanoparticles when the field is active. More importantly, the strength and direction of the external magnetic field govern the direction and extent of their elongation.

The current study shows the viability of a novel site-specific drug delivery approach based on the uniform-field-induced magnetization effect and its application for drug-loaded magnetic carriers targeting stented arteries.

The benefits of our flow phantom configuration concerning the external magnetic field are as follows: (1) allowing for relatively high local magnetic gradients deep within the human body; (2) allowing for multiple, targeted drug delivery and consequently treatment modalities using handy systemic drug injections (i.e., repeated targeted drug delivery in cancer therapy); and (3) the targeting procedure’s reproducibility for the same flow parameters.

We are confident that our findings on site-specific placement in the vasculature utilizing a combination of magnetoresponsive particles and magnetic fields will contribute to improving vascular disease experimental therapies.

As reported in our earlier investigations, blood smear, blood sedimentation rate, white blood cell viability examinations, and interaction tests of PEG_MNP with human plasma [22] indicated the theranostic potential and the hemocompatibility of the PEGylated MNPs. Additionally, PEGylated MNP can freely flow across the vascular system and be directed to a particular site-specific region via magnetic drug targeting processes, as demonstrated in our previous work [57].

## Figures and Tables

**Table 1:** Rheological characterization of the model suspensions.

Fluid	*T* [°C]	*B* [mT]	*η*_∞_ [Pas]	*η*_0_ [Pas]	*C* [s]	*p* [-]	*r^2^*
CF + 0.1% PEG_MC	25	0	0.00154	0.095	18.08	0.664	0.999

Where: *T* [°C] is the carrier fluid temperature, *B* [T] is magnetic field intensity, *η*_∞_ [Pas] is the viscosity at infinite shear rates, *η*_0_ [Pas] is zero shear viscosity, *C* [s] is the characteristic time constant, and *p* [-] flows behaviour index. The *r*^2^ values for all fits are close to unity, indicating an excellent fit (*r^2^* is the coefficient of determination used to evaluate the quality of the Carreau fits).

**Table 2:** The values of the fit parameters were obtained by fitting the viscosity curves with the Carreau model for different magnetic flux density values.

*T* [°C]	*B* [mT]	*η*_∞_ [Pas]	*η*_0_ [Pas]	*C* [s]	*p* [-]	*r^2^*
25 °C	0	0.001	0.182	7.645	0.559	0.986
	42	0.001	86.6	12.20	0.478	0.996
	52	0.001	98.00	12.31	0.486	0.994
	117	0.001	155.35	17.00	0.513	0.996
	183	0.001	322.20	21.26	0.517	0.991

Where: *T* [°C] is the carrier fluid temperature, *B* [T] is magnetic flux density, *η*_∞_ [Pas] is the viscosity at infinite shear rates, *η*_0_ [Pas] is zero shear viscosity, *C* [s] is the characteristic time constant, and *p* [-] flows behaviour index. The *r*^2^ values for all fits are close to unity, indicating an excellent fit (*r^2^* is the coefficient of determination used to evaluate the quality of the Carreau fits).

**Table 3:** Investigated chain length variations.

Time [s]	Chain 1 [mm]	Chain 2 [mm]	Chain 3 [mm]
0 (start)	0	0	0
10	0.26	0.47	0.21
20	0.84	0.58	0.27
30 (end)	0.86	0.63	0.27

## Data Availability

Not Applicable.
